# Issues affecting supply of palliative medicines into community pharmacy: A qualitative study of community pharmacist and pharmaceutical wholesaler/distributor perspectives

**DOI:** 10.1016/j.rcsop.2022.100132

**Published:** 2022-04-04

**Authors:** Natasha Campling, Liz Breen, Elizabeth Miller, Jacqueline Birtwistle, Alison Richardson, Michael Bennett, Susan Latter

**Affiliations:** a School of Health Sciences, University of Southampton, Building 67, Highfield, Southampton SO17 1BJ, England, UK; b University of Bradford School of Pharmacy and Medical Sciences, M27a Richmond Building, Richmond Road, Bradford BD7 1DP, England, UK; c St Luke's Hospice, Little Common Lane, Sheffield S11 9NE, England, UK; d Leeds Institute of Health Sciences, University of Leeds, Worsley Building, Clarendon Way, Leeds LS2 9NL, England, UK; e University Hospitals Southampton NHS Foundation Trust, Southampton General Hospital, Tremona Road, Southampton SO16 6YD, England, UK

**Keywords:** Supply, Community pharmacy, Pharmaceutical wholesalers/distributors, End-of-life, Medicines access, Palliative care medicines, CD, Controlled Drug (controlled substances), CP, Community Pharmacist, EoL, End-of-Life (pertaining to the last year of life), FL, Full-line wholesaler, GP, General Practitioner (family doctor), I, Independent pharmacy, LM, Large multiple pharmacy, SL, Short-line wholesaler, SM, Small multiple pharmacy, WD, Wholesaler/distributor

## Abstract

**Background:**

Patient access to medicines in the community at end-of-life (pertaining to the last year of life) is vital for symptom control. Supply of such medicines is known to be problematic, but despite this, studies have failed to examine the issues affecting community pharmacy access to palliative medicines.

**Objective:**

To identify community pharmacists' and pharmaceutical wholesalers'/distributors' views on supply chain processes and challenges in providing access to medicines during the last year of life, to characterise supply in this UK context.

**Methods:**

Qualitative design, with telephone interviews analysed using Framework Analysis. Coding frames were developed iteratively with data analysed separately and then triangulated to examine differences in perspectives.

**Findings:**

Thirty-two interviews (24 community pharmacists and 8 wholesalers/distributors) were conducted. To ensure appropriate palliative medicines were available despite occasional shortages, community pharmacists worked tirelessly. They navigated a challenging interface with wholesalers/distributors, the Drug Tariff to ensure reimbursement, and multiple systems. IT infrastructures and logistics provided by wholesalers/distributors were often helpful to supply into community pharmacies resulting in same or next day deliveries. However, the inability of manufacturers to predict operational issues or accurately forecast demand led wholesalers/distributors to encounter shortages with manufactured stock levels, reducing timely access to medicines.

**Conclusions:**

The study identifies for the first time how palliative medicines supply into community pharmacy, can be improved. A conceptual model was developed, illustrating how influencing factors affect responsiveness and speed of medicines access for patients. Work is required to strengthen this supply chain via effective relationship-building and information-sharing, to prevent patients facing disruptions in access to palliative medicines at end-of-life.

## 1 Introduction

The pharmaceutical supply chain exists to ensure medicines are supplied to patients within time and financial tolerances.^1^ However, this particular supply chain is known for its complexity,^2^^,^^3^ encompassing tiers of operations in a convoluted network that facilitates manufacture and distribution.^4^ As a result of this convolution, extensive lead times and unpredictable production (in response to demand), fragility is often evident.^5^ This fragility has been highlighted during the COVID-19 pandemic,^6^^,^^7^ where there has been immense pressure to maintain continued supply of medicines during a challenging period in global healthcare provision. Whilst the pandemic has created product shortages, this is not a new phenomenon.^7^, ^8^, ^9^, ^10^, ^11^, ^12^, ^13^, ^14^, ^15^ The body of literature has focused on pharmaceutical supply chain management (for example^4^^,^^5^), global medicines supply and issues of availability, particularly medicines shortages.^8^, ^9^, ^10^, ^11^, ^12^, ^13^, ^14^, ^15^, ^16^, ^17^ However, research to date has focused on types and impacts of supply issues as experienced by hospital pharmacy in the main. There is a lack of literature focused on supply into community pharmacy specifically and the views of community pharmacists as recipients of medicines supply and pharmaceutical wholesalers/distributors as providers of these products. Yet community pharmacy and pharmacists are a critical element of the patient-facing supply chain in the UK, significantly influencing patient’ ability to access to medicines.

In the UK community (retail) pharmacies range from large multiples (LM) >100 pharmacies) with chain stores, through to small multiples (SM) 6–99 pharmacies) and individually owned pharmacies, independents (I) 1–5 pharmacies) in small communities.^18^^,^^19^ Between 2018 and 2019 there were 11,539 community pharmacies in England, each dispensing on average 87,212 items of which 64.9% were via the Electronic Prescribing Service (EPS).^20^ Analysis states that LMs comprise 49.4% of the English, Welsh and Scottish market, followed by independents at 37.3% and SMs at 13.3%.^18^ Pharmaceutical distributors purchase medicines from pharmaceutical manufacturers for storage in warehouses and distribution centers.^21^ Community pharmacies place orders with distributors for the medicines they need, and the distributors process and deliver the orders. Of these distributors, full-line (FL) wholesalers carry and distribute a wide variety or complete range of pharmaceutical products. They handle large sales volumes and provide a broad range of services such as stocking inventories, extended financing services (supplying credit) and employ telesales staff.^22^^,^^23^ In contrast, short-line (SL) wholesalers offer a limited range of products, mainly generics and some parallel imports, cutting costs by limiting the medicines available, only supplying medicines where sufficient margins can be made.^22^

Local community pharmacies are used by patients in England to obtain their medicines; community pharmacists dispense medicines to people living in their locality and there is a national policy drive for them to play a greater role in patient care, including palliative care.^24^ Supply into community pharmacy is pivotal to patient access to palliative medicines for those living at home at the end-of-life (EoL), with such medicines critical to both symptom control and quality of life, yet it is known to be problematic in practice. Indeed, the COVID-19 pandemic has led to increased numbers dying at home requiring palliative medicines. In the UK there was a sustained increase in the number of people dying at home in all four nations throughout 2020, with a further increase during the second pandemic wave.^25^ Despite this increase in need, and the likelihood of a sustained increase in those dying at home over the next decade, it is known that supplies to patients are hindered by community pharmacy-held stock levels of palliative medicines.^26^ In a study of factors causing delays in medicines access for palliative care patients under the care of a specialist home care provider in Ireland, palliative care specialist nurses reported instances of delayed supply of which half were due to medicines not being in stock in community pharmacies.^27^ The study found that in a survey of 57 community pharmacists half (49%) reported some palliative medicines were not stocked and a third had difficulty in obtaining supplies. Miller in a small-scale study of community pharmacies in the North of England providing palliative medicines also demonstrated stock to be an issue, leading to delays in patient access.^28^ Lack of access to pharmacy stock was similarly cited as problematic by healthcare professionals providing community palliative care services in a study exploring gaps in service provision in Australia.^29^ More recently, in an England-wide survey of 219 community pharmacists 76 (35%) sometimes, 79 (36%) often, or 14 (7%) always encountered a discrepancy between palliative medicines prescribed and the stock they held.^30^ Thirty-eight (18%) sometimes, 41 (19%) often, or 15 (7%) always limited their stock of palliative medicines because of storage space. Lastly, 67 (31%) often, 47 (22%) sometimes, or 40 (18%) always limited their stocks of palliative medicines due to concerns regarding expiry dates.

Accessing supplies of palliative medicines in the community may prove burdensome and distressing for patients and their caregivers as the patient deteriorates due to symptom changes, necessitating frequent changes in the range and formulations of medicines prescribed. In addition, supply at EoL is complicated by regulations associated with the stock and supply of controlled drugs (CDs), which form a significant proportion of commonly used palliative medicines. This context is thus indicative of the significance of potential supply issues, where supply needs to be responsive to the rapidly changing, urgent needs of individuals dying at home. Given this difficulty, combined with a lack of research focused on supply of medicines into the unique and important setting of community pharmacy, the authors sought to evaluate supply in this context by interviewing community pharmacists (CPs) and pharmaceutical wholesalers/distributors (WDs). To fully understand the complexity of this supply chain a whole systems perspective was adopted. This encompassed examining how groups within the supply chain interrelate (influenced by information transfer and professional perspectives): WDs and manufacturers (from the perspective of WDs); CPs and manufacturers (from the perspective of CPs); WDs and CPs (from the perspective of both sample groups); and CPs and patients/carers (from the perspective of CPs).

Data reported here formed part of a larger study (ActMed) evaluating Access to Medicines for patients at EoL in the context of service delivery characteristics. For further details see: https://www.journalslibrary.nihr.ac.uk/programmes/hsdr/165223/#/. The study has been fully reported to the National Institute for Health Research (November 2020) and will be published in their Journals Library following peer review.

## 2 Methods

A qualitative study design was taken; telephone interviews were sought with 20 CPs and 10 WDs to facilitate data saturation. The qualitative design was underpinned by a whole systems perspective to characterise the complexity of the supply chain, examining how various systems and influencing factors within the supply chain interrelate. Standards for reporting qualitative research (SRQR)^31^ have been utilised.

### 2.1 Sampling

CPs were purposively sampled via all 15 Clinical Research Networks (these networks support healthcare organisations and individuals to participate in high-quality research) in England. Key contacts within these networks distributed the invite to CPs (via research active community pharmacies, pharmacist leads or champions, and/or community pharmacy networks) and distributed one reminder six weeks later (spanning July–September 2019). Additionally, some CPs were recruited via another phase of the larger study in which CPs participated in interviews about their specialist provision of palliative medicines services. Snowball sampling also occurred where CPs gained agreement from colleagues to be approached. CPs were sought from community pharmacies across Clinical Research Network regions.

**Fig. 1:**
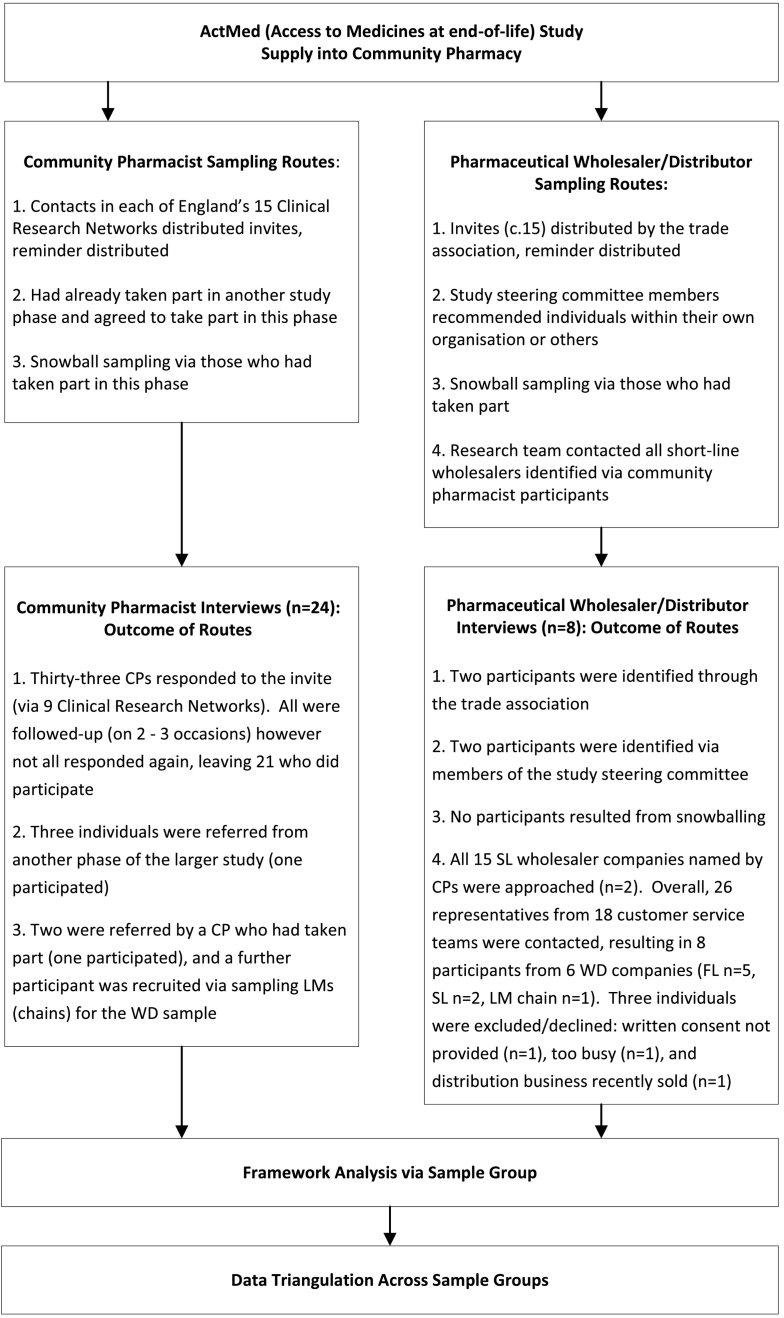
Sampling, data collection, and analysis.

For WDs a range of approaches were utilised to purposively sample ‘elites’, those with decision-making responsibility at senior management and board level.^32^ Four routes to sampling were used to target FL and SL wholesale participants, and members of the trade association representing WDs with a distribution or wholesale role within LMs (chains). Potential participants were excluded if their expertise focused solely on hospital not community supply. Refer to Fig. 1 for further details.

### 2.2 Data collection

Semi-structured interviews were conducted. The study was introduced to the interviewees by outlining the focus of the wider study on patient and carer experience of access to medicines at end-of-life, and explaining that this phase of the study was focused on interviewees' experiences of supply processes (from a whole systems perspective, how elements of the entire supply system interrelate – considering relationships, information flows, and professional perspectives) and any challenges in providing access to medicines used for symptom management during the last year of life.

Interview guides were developed by five members of the research team (NC, AB, SL, EM and LB), informed by a systematic literature review,^26^ emergent findings from other study phases (an online survey of healthcare professionals; and case studies), and study steering committee meeting discussions. Data were sought on issues such as: roles played in facilitating access to palliative medicines; experiences of supply chain processes including barriers and facilitators to supply; communication and information transfer between sample groups; and influences on, and related decision-making about, stock and supplies.

### 2.3 Data analysis

Following informed consent all interviews were audio-recorded, fully transcribed and analysed using Framework Analysis via the following phases: familiarisation, identifying a framework, indexing, charting, and mapping and interpretation.^33^ Coding frameworks for each sample group were developed by four members of the research team (NC, EM, LB and SL). The first three interviews in each sample were independently coded by three members of the team (NC, LB and EM). Coding was discussed with differences resolved, and coding frameworks iterated. Interviews with CPs and WDs were analysed separately and then triangulated to examine differences/similarities in perspectives.

### 2.4 Ethical considerations

Research ethics approvals were obtained from the NHS Health Research Authority South Central – Hampshire A Research Ethics Committee (REC reference: 18/SC/0675).

To summarise, Fig. 1 displays the sampling, data collection and analysis processes used in the study.

## 3 Findings

Twenty-four interviews with CPs and 8 interviews with WDs were undertaken (*n* = 32 overall). CP interviews ranged from 14 to 38 min (median 30), WD interviews from 17 to 30 min (median 27).

### 3.1 Community pharmacist sample

Table 1 displays the CP sample. Twenty-four CPs were interviewed August–September 2019 by NC (an experienced qualitative researcher with a palliative care clinical background). Participants stated the number of WDs used and a best approximation reflecting participants' knowledge and recollection. Between 1 and 3 FL wholesalers were utilised by participants in their community pharmacies (median 3). Some pharmacies (LMs) used no SL wholesalers whilst others used up to 14 (median 3), for others this was difficult to estimate as they utilised the services of a third-party to place orders via SL wholesalers. Fifteen SL wholesalers were named by participants as being used. Overall, 2–16 wholesalers (both FL and SL) were used (median 5).

**Table 1:** Characteristics of community pharmacist sample.

Total number of pharmacies	24
Size of pharmacy^a^	
Independent	11
Large multiple	7
Small multiple	6
Provision of commissioned service for palliative care by pharmacy^b^	
Providing commissioned service	5
Not providing commissioned service	19
Location of pharmacy via Clinical Research Network region	
North West London	7
Eastern	4
North West Coast	3
Kent, Surry & Sussex	2
East Midlands	2
Greater Manchester	2
South London	1
North East and North Cumbria	1
Thames Valley and South Midlands	1
Yorkshire and Humber	1
Range in number of prescriptions dispensed per month by pharmacy (Sept 2019)^34^	1469–16,918
Wholesaler/Distributor usage by pharmacy	
Range in number of full-line wholesalers used	1–3, median 3
Range in number of short-line wholesalers used	0–14, median 3
Range in overall number of wholesalers/distributors used	2–16, median 5

a Pharmacy size classification – Large multiples >100 pharmacies, small multiples 6–99 pharmacies, independents 1–5 pharmacies. Total United Kingdom market - large multiples 49.4%, independents 37.3%, small multiples 13.3%.^18^

b Community pharmacy-delivered commissioned services for palliative care are funded to provide locally or regionally determined stocks of “core” lists of palliative medicines and community pharmacy extended hours of opening where possible.

### 3.2 Wholesaler/distributor sample

Eight WD interviews were undertaken May–November 2019 by EM (a qualitative researcher with palliative care pharmacy expertise). The 8 participants were from 6 WD companies (FL *n* = 5, SL *n* = 2, LM chain *n* = 1).

**Fig. 2:**
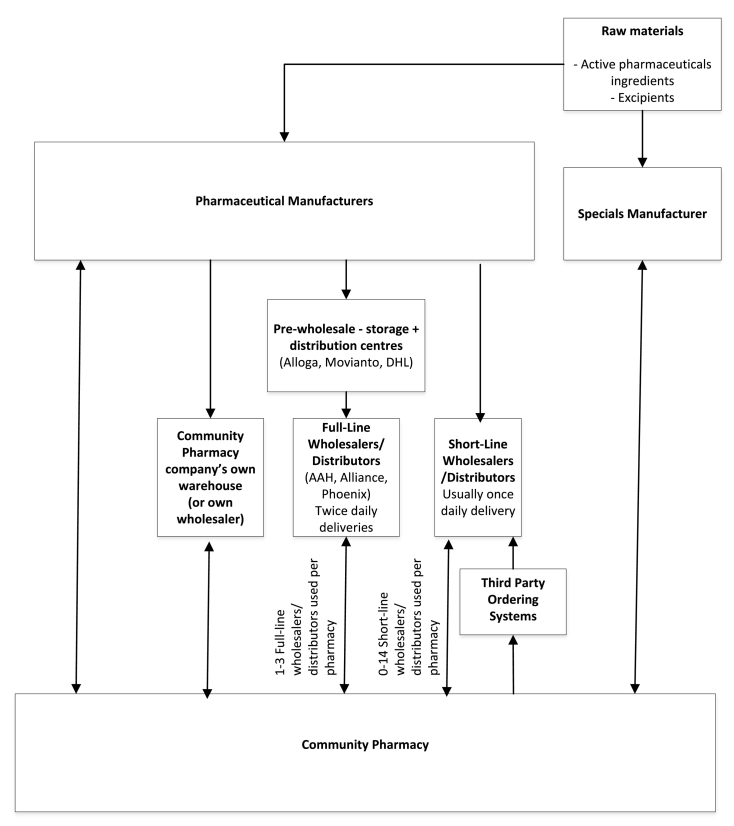
Supply chain routes into community pharmacy.

The study findings are presented below, for further quotes see supplementary data file 1 (Table of quotes). Fig. 2 illustrates supply routes into England's community pharmacy generated from study data.

## 4 Community pharmacist findings

### 4.1 Role played facilitating medicines access

CPs provided insight into their role facilitating medicines access at EoL. This encompassed: stock management within their pharmacy, or across numerous pharmacies; anticipating and/or triaging prescriptions for patients; dispensing of medicines to a patient/family member; providing information about medicines and how to access them to patients/family members; provision of home delivery; and where commissioned, provision of a palliative medicines service (see Table 1). Pharmacist roles were helped by experiential knowledge, some practicing for over 20 years and holding senior positions.

CPs perceived medicine supply to those in the last year of life to be central to their role. It was often an emotive issue for them:

*“…These are the most vulnerable people at the most vulnerable time looking to spend time [together] at the end of their life and a prescription is no use to them nor their family…”* (CP16, I).

*“…All they (family members) want to do is come and pick their loved ones medication up. So in many cases I don't even try to explain I just do my damnedest to get hold of it…”* (CP15, I).

To ensure continuity of medicine supply to patients, some participants pre-emptively engaged in stock management:

*“…We generally take a proactive approach to medicine stock holding… we'd rather be looking at it than looking for it… we carry a significant range of controlled drug medicines and medicines that might be required in end-of-life situations…”* (CP10, I).

Other CPs reported acting pre-emptively on behalf of patients, anticipating prescriptions being sent electronically (via the Electronic Prescription Service) to the pharmacy, to facilitate supply; whilst others discussed how they triaged (and prioritised) such prescriptions.

CPs emphasised that to fulfil their role facilitating medicines access, they were reliant on building relationships with patients, families, and healthcare professionals. Often embedded in their communities by virtue of them being well-established businesses, CPs sought to facilitate seamless care. Some had developed strong relationships with patients and their families over numerous years. To provide medicines access and necessary services to patients, some CPs worked to develop and maintain relationships with a myriad of relevant healthcare professionals (local GPs, community-focused palliative care nurse specialists, community nurses, and other pharmacists) with varying levels of success.

“…*Specialist nurses, community nurses, community matrons… all call in to us, we supply them, we help them, we deliver, we call out. We do as much as we can to help them… There are strong relationships with those people...”* (CP16, I).

“…*I personally know all the GPs… because I've been around for so long… We have got a GP who is a lead palliative care GP across the area and she's in my local practice and she and I have got a fantastic relationship. We'll text each other; she'll text me and say I've got someone who is going to need X and Y can you sort it…?*” (CP21, LM).

### 4.2 Facilitators of supply into community pharmacy

#### 4.2.1 Use of key wholesalers/distributors

CPs spoke of using key WDs for supply into their pharmacies. Pharmacies were likely to use one, occasionally two, WDs as first-line options. Using key WDs could facilitate communication and relationships between the pharmacy and WD. For some pharmacies, who had their own wholesaler (i.e. owned by the respective SM or LM), supply was facilitated. Most CPs discussed their, or their employing company's, prioritisation of WDs i.e. which they used in which circumstances (first, second, and consecutive options). Decisions concerning which WDs to engage with hinged on cost (best price), availability, and speed of supply.

#### 4.2.2 Information technology systems (Stock Management and/or Ordering)

Supply into community pharmacy was facilitated by IT systems for stock management within pharmacies and for placing orders with WDs. The extent to which pharmacies used IT systems to manage their stocks varied but all pharmacies placed orders with WDs via online systems. Only a few SL wholesalers did not provide any online ordering platform. Generally, online ordering systems were perceived to facilitate supply.

#### 4.2.3 Time to delivery

Another facilitator of supply into community pharmacy was time to delivery. CPs were largely satisfied with the time to delivery offered by WDs. FL wholesalers were able to provide twice daily deliveries, and SL wholesalers provided once daily delivery (with some able to provide twice daily deliveries). For many CPs there were multiple deliveries per day because of their use of numerous WDs.

CPs emphasised that where orders needed to be placed to fill a prescription, if WDs had their own stocks then delivery could be that same day (weekdays) for orders placed before the respective cut-off time. Once the cut-off time had been crossed then delivery into the pharmacy would be for the following day. Ability of WDs to provide same or following day delivery was perceived to be as good as possible, considering the need for supplies to be transported from distribution centres/warehouses. Where third parties were used then supply would be for the following day.

#### 4.2.4 Sourcing stock from other pharmacies

On occasion where pharmacies did not hold stocks of the required medicines, nor could source them via WDs, most CPs contacted other pharmacies on behalf of patients. They spoke of contacting nearby pharmacies that were part of LMs because of their likelihood of holding more extensive stocks within store, and their ability to contact other branches within the respective chain. Most CPs referred to use of community pharmacy networks in sourcing medicines from other pharmacies, often run via instant messaging apps.

### 4.3 Barriers to supply into community pharmacy

For CPs barriers to supply into community pharmacy, and ultimately medicines access, outnumbered facilitating factors. This did not mean patients necessarily had poor experiences of community pharmacy-related medicines access, rather there were numerous hurdles that needed to be overcome, and CPs worked tirelessly to overcome these.

#### 4.3.1 Medicine shortages

General medicine shortages were a universal challenge for CPs. Lack of information surrounding medicines shortages was problematic with CPs having to seek information via various sources (e.g. professional organisations, WDs and manufacturers). This contributed to pharmacist workload, and further delays in accessing medicines. For palliative medicines specifically, CPs reported varying levels of difficulty related to shortages *“which is more distressing...”* (CP07, I) but *“…from the palliative care point of view and we haven't experienced the problems that we've seen in other parts of our business with non-availability of drugs generally…”* (CP12, SM). It appeared that for a select few (with interest and specialism in this area) they successfully weathered any shortage issues because of effort put into sourcing medicines, sometimes from all over the world.

General medicine shortages also led to quotas being imposed by WDs and/or manufacturers. These were perceived as a hurdle which CPs had to navigate to gain supplies. They were often harshly viewed by CPs as creating additional work and supply delays to patients.

*“…They give you so many for the month, if you use any more than that tough ‘you can't have them, they're quota'd’. You have to then mess around to try and get an extension on the quota, which involves faxing prescription copies to them to prove that you've got a prescription for that item, which again is so time consuming and a complete waste of time...”* (CP14, I).

Where medicines became short in the market, some CPs believed that WDs and manufacturers prioritised hospital supply, further limiting supply into community pharmacy.

Some palliative medicines were in short supply during the data collection period but this was not a frequent occurrence, which was not the case for other medicines where shortages were more common. When palliative medicines were in short supply CPs highlighted that any shortage in the market would drive up the price of the medicine. This was a key issue due to professional obligations to supply the medicines, but prices frequently exceeded the monthly stated Drug Tariff price (i.e. purchase cost to the pharmacy exceeds reimbursement price). At the time of purchase CPs did not know whether a price concession (products listed monthly by the Government's Department of Health and Social Care where the medicine is available above the set Drug Tariff reimbursement price and is reimbursed at the concession price rather than the Drug Tariff price) would be granted retrospectively and at what price. This led to negative consequences, with pharmacies dispensing such medicines at a loss, contributing to some pharmacies operating at a loss overall. Alternatively, medicines were returned to WDs because the purchase price was deemed too far in excess of the Drug Tariff price, leading to delays in patient access.

Where medicine shortages occurred, CPs perceived placing a request for a prescription change via the prescriber as “*last resort*”. It was apparent that getting a prescription changed via the prescriber contributed to delay in medicines access for the patient.

#### 4.3.2 Need to use multiple wholesalers/distributors

Although utilising key WDs (see ‘use of key wholesalers/distributors’) was a facilitator to supply, conversely the need to use multiple WDs acted as a barrier to supply. This precluded straightforward supply, adding complexity to supply chain routes into pharmacies. It also contributed to the onerous CP workload as they dealt with numerous WDs and/or third parties.

*“…We work with all the wholesalers, we have to to get supplies. It's very tricky at the moment, there are lots of supply issues so we're shopping around from one to another…”* (CP14, I).

The necessity to use multiple WDs was part of the context of medicine shortages. CPs had to increase numbers of WDs to accommodate shortages. CPs also perceived that Solus agreements (where the manufacturer uses a sole/single WD to distribute their products); contributed to their need to use numerous WDs. They were often critical of these contracts, describing them as monopolies or restrictive practices. For some CPs (mainly those in independents) it was only their use of numerous WDs that enabled them to accommodate such Solus agreements and access the full range of medicines prescribed for patients during the last year of life.

#### 4.3.3 Lack of communication and relationships with wholesalers/distributors and manufacturers

Another barrier to supply was the lack of meaningful communication (two-way information transfer underpinned by trust) with WDs and manufacturers, and the consequent lack of relationships. CPs highlighted that when they needed to speak to WDs, they did so by telephoning the respective company's customer services team. It was relatively rare (according to CPs) for WDs to contact CPs, so communication was generally pharmacist-initiated. Ringing service centres was time-consuming, like ringing a “*call centre*”, not knowing who they were talking to.

The lack of clinical insight held by those answering the phones at WDs was an issue for some CPs because they did not understand the urgency of palliative care medicine supply. Furthermore, a lack of understanding could preclude WDs' sales staff searching for alternative options and total reliance on their IT systems. Level of insight was not just an issue for customer service teams at WDs. It could be confounded by CPs delegating calls to dispensers. This meant that they were less likely to consider the following options:

*“…If one thing is not available I might… say ‘is it available as a slightly different formulation, a slightly different strength’… then I can know that I can go back to the prescriber quickly and say ‘I can get this, can you please change the prescription to X…’”* (CP09, LM).

This lack of meaningful communication precluded relationship development between CPs and WDs and was a fundamental barrier to supply.

“…*I don't think you do have a relationship with them, not like you used to. There are no reps that come round. I wouldn't even know who my account managers were anymore with these big companies [FL wholesalers]. Never see them. Never ring up or anything…”* (CP14, I).

Lack of meaningful communication and relationships appeared to be underpinned by mistrust on the part of the CPs towards WDs and manufacturers, triggered by conflicting cultures and priorities. CPs argued they were focused on patient needs (accountability to the patient), whilst they perceived WDs and manufacturers to be focused on commercial priorities.

*“…[To get access to medicines]… I have to jump through hoops, spend time which equals money, takes me away from looking after patients and all of my staff away from looking after patients to try and source medication or products… they're commercial operations looking to make the most that they can out of what they're doing...”* (CP16, I).

#### 4.3.4 Shortcomings of ordering systems

Despite IT ordering systems being a facilitator to supply (see ‘information technology systems’), many CPs identified shortcomings of online systems conversely acting as a barrier. CPs stated that the majority of orders could be dealt with solely online, but as soon as an issue arose such as a product being identified as out of stock with the WDs, they would need to ring the WD to find out if/when it would be back in stock. Other reasons for needing to phone were to find out about: specific brand availability; price of the product (as prices altered daily); where a product was low in stock if they actually had it; if the product was a switch line (switched to a different warehouse/distribution centre and therefore how long it would take to be delivered); and where a third-party order platform was used to ascertain availability (as product availability was not stated on these platforms). The phoning round, although it did not occur for most ordering, was perceived as hugely time-consuming to gain “*definitive answers*” and source product for the patient.

Some WDs were viewed as having more live and robust online ordering systems than others, but examples provided were frequently negative:

*“…One of the [FL] wholesalers will accept the order, not tell you anything is out of stock and then it doesn't come in and then you ring them … they say, ‘well it's out of stock’. ‘Well why is your computer?’ ‘Oh, I don't know’, is the answer...”* (CP21, LM).

Therefore, although online ordering systems were primarily seen as a good thing, they were viewed as limited in functionality.

#### 4.3.5 Disincentives to stocking palliative medicines

Another barrier to supply was disincentives to stocking palliative medicines. Pharmacies from which CPs operated varied widely in the number of prescriptions dispensed per month (refer to Table 1), so for some, a lack of turnover of palliative medicines was a strong disincentive for stocking such medicines.

*“…The hurdle is that the medications are usually high value, so pharmacists don't tend to keep them in stock. They're also not commonly prescribed, you don't know which strength they're going to be...”* (CP17, LM).

Related to this potential lack of stock turnover were the associated costs of the medicines and the lack of a long shelf-life for some (only those operating commissioned services for palliative care were reimbursed for expired medicines, refer to Table 1. These were services funded to provide locally or regionally determined stocks of “core” lists of palliative medicines and community pharmacy extended hours of opening where possible). CPs could also be concerned of the risk of medicines not being collected by patients or their families.

Some CPs discussed disincentives specific to stocking CDs often used in palliative care. These were: legal requirement to store in locked cupboards (and limited CD cupboard capacity); inability to return CDs to WD where the medicines were not collected by the patient/family; and requirements around destruction of out-of-date CDs. In the main, disincentives revolved around implications, including cost, of medicines not being collected. Disincentives to stock CDs did appear to influence thinking and behaviours of some CPs, meaning sufficient CDs were less likely to be held in stock than other medicines.

#### 4.3.6 Lack of weekend ordering and sunday deliveries

CPs who worked at weekends emphasised that supply over weekends could be an issue for patients. Although pharmacies usually had one delivery (per WD) on a Saturday the inability to place orders over the weekend, and the requirement to wait for Monday's deliveries was a barrier to supply into pharmacies, and ultimately medicines access over the weekend.

#### 4.3.7 Issues with wholesaler/distributor deliveries

Some CPs referred to issues such as road accidents precluding deliveries. Usually, they were described as an occasional occurrence. For others delivery issues appeared more frequent, particularly in relation to missing products from an order which were not known until the delivery arrived.

## 5 Wholesaler/distributor findings

### 5.1 Role played facilitating medicines access

WDs discussed strategic elements in supplying palliative medicines to community pharmacies. These comprised commercial and quality ‘*value-added*’ services to win community pharmacy business from WD competitors through use of Solus contracts and quality improvement of facilities and infrastructure to improve WD volume of stock in the marketplace. Three national wholesalers provided a FL service of all pharmaceuticals including palliative medicines, in contrast SL wholesalers provided a partial range usually at a competitive price. Competition in the branded medicines market was more limited than within the generic market with the three FLs competing for manufacturers' business. Some participants discussed Solus or dual arrangements which according to WDs assured continuity of supply in the market due to WD close relationship with manufacturer. These arrangements were also perceived to generate more secure business.

“…*Commercially it's better for us to get all of the volume where you get 100% market share [Solus contract]. We're in a volume-based business so this brings us volume...”* (WD03, FL).

Despite this perception, Solus contracts were reported by SLs as increasing the risk of supply failure since the product could not be accessed via other WDs. Commercial drivers also dictated the discount awarded to pharmacies based on the volume of stock purchased from WDs. However, within Solus contracts there was arguably less need to be competitive, being the sole supplier.

Inability to secure sufficient stock levels was considered a commercial ‘faux pas’ as it had a negative impact for the business as a commercial enterprise, not having assets to sell, but also not being able to offer the expected service to CPs. WDs struggled to differentiate within the market, offering very similar services and so needed to differentiate in other ways, stock availability to ensure service continuity potentially being one of these. Quality improvement of customer service, IT ordering, storage facilities and logistics infrastructure were reported as key means to maintain a WDs' competitive position and increase the volume of stock sold.

Strategic improvement and control of the supply chain were also seen as priorities as there was less risk attached to a streamlined supply chain with fewer companies in the sequence of activity, enabling *“a very tight and secure, assured supply chain”* (WD05, FL). Regular auditing of partners and by the Government's medicines regulatory agency also ensured WDs focused on regulatory compliance and patient safety. In addition, working with pharmacies to respond to their service complaints was important as complaints challenged working relationships.

### 5.2 Facilitators of supply into community pharmacy

#### 5.2.1 Relationship-building

WDs noted the importance of relationship-building in facilitating medicines access whether through formal agreements or contracting arrangements with manufacturers, day-to-day relationships with community pharmacies (via sales and customer service teams) or informal communications and networks. Relationships were stated to support regular dialogue with a two-way flow of information and feedback on problems up and down the supply chain.

#### 5.2.2 Upstream relationships with manufacturers/suppliers

Contracts or agreements with manufacturers/suppliers could provide assurance of inbound stock to WDs, however formal contracts were not universally used due to manufacturers/suppliers not being able to guarantee supply against an order e.g. adverse weather, shortage of raw materials or quality audit failure. Due to this reason, WDs collaborated closely with manufacturers/suppliers often through designated account managers to try and assure product availability. When supply disruptions were envisaged, manufacturers were responsible for communicating this to the Department of Health and Social Care (a Government department responsible for policies on health and social care in England).

#### 5.2.3 Downstream relationships with pharmacies

WDs reported community pharmacies accessed information on medicines shortages from on-line ordering systems and customer services teams. This was viewed as two-way conversation, with telesales representatives providing feedback to WDs and manufacturers. WDs reported they had a strategic role to put pressure on manufacturers when shortages were identified by pharmacies. They asserted they benefitted from their role in this triad by transferring information from pharmacies upstream to manufacturers e.g., regarding changes in prescribing patterns. This could instigate proactive responses from manufacturers regarding production plans/stock level holding.

#### 5.2.4 Collaborative relationships

Some WDs discussed good practice in supply chain management where severe (high impact) medicines shortages, such as with diamorphine 5 mg injection, had been co-ordinated nationally via the Department of Health and Social Care's Medicines Supply Team and NHS England & NHS Improvement's Commercial Medicines Unit. In such situations, there was a willingness to work collaboratively to get medicines to patients, setting aside competitive relations in response to the shortage.

Relationships between WDs, manufacturers and community pharmacies were viewed as extremely important in supplying palliative medicines, WDs reported acting as a point of mediation in the supply chain.

#### 5.2.5 Investment in logistics infrastructure

WDs identified logistical issues in the pharmaceutical supply chain as critical in ensuring medicines access at EoL. There was emphasis on the requirement to deliver on time and in full so they could be responsive to community pharmacy and patients' needs. This was facilitated by contracting with reputable haulage firms familiar with regulatory governance, investing in logistics infrastructure and by having clear visibility of stock levels.

WDs reported high-quality logistics infrastructure ensured stock could be delivered with increased certainty, and orders delivered on the same or next day, on time and complete. Some participants across FL and SL wholesalers discussed the value of twice daily deliveries to community pharmacies in expediating medicines access.

*“…Wholesalers deliver stock eleven times a week to pharmacies…so actually what a wholesaler does is provide access…we deliver twice a day and every weekday to all of those customers...”* (WD03, FL).

#### 5.2.6 Demand and stock management

WDs sought to ensure supply continuity into community pharmacy. A key element in providing medicines to community pharmacies was WDs having access to stock within the UK market, or if a shortage, their ability to source alternative product elsewhere (outside of the UK). Where the product was generic there tended to be a greater source of alternative suppliers. If a product could not be sourced, it was because it could not be (as opposed to no attempt made).

*“…Majority of time that shortages occur are about not having the product available for supply and that's down to maybe raw material, maybe choice and allocations to different countries, maybe production issues…”* (WD05, FL).

*“...If we can't get hold of one drug, we'd probably work with another company that had got a competitor drug...”* (WD02, FL).

WDs advised they shared their demand profile and activity with manufacturers, to inform manufacturing capacity management. This information transfer aimed to ensure stock levels were as needed, and medicines shortages did not develop.

The majority of WDs described complex systems for managing stock in response to forecasted demand. Stock management was crucial to WDs' ability to supply medicines. WDs considered stock issues could occur through ineffective capacity planning (manufacturer) or excessive demand (pharmacy). Most medicine shortages were perceived to derive from insufficient ‘inbound’ stock from manufacturers, but problems could occur when demand from pharmacies exceeded supplies, leading WDs to introduce quota systems to ration medicines.

*“…Unfortunately, the stock that you may require is finite in quantity, you may not always get as much as you forecast you may need…”* (WD08, SL).

Intelligence regarding customer demand patterns, stock holding levels and locations of stock meant WDs could adjust stock levels throughout the country in distribution centres to respond to demand spikes e.g. where stock was switched at a regional scale causing other products to be in demand.

#### 5.2.7 Buffer stock availability

Most participants discussed how buffer stocks, stocks within the UK and Western Europe, had an important role in adding resilience to the supply chain to facilitate medicines access. Holding buffer stock could be recommended by the manufacturer to the pre-wholesaler if they expected a product shortage or it could involve the WD transferring stock between distribution centres (ensuring quicker response times for orders and equitable distribution).

WDs reported that stock availability was always dependent on manufacturer production schedule and lead time for distribution. SL wholesalers were noted to fill a gap when FL wholesalers were devoid of stock to maintain supplies into community pharmacies.

### 5.3 Barriers to supply into community pharmacy

WDs discussed barriers to supply of palliative medicines which were often outside of their control. Such barriers included: supply chain disruptions caused by product shortages or recalls; manufacturer strategic drivers such as manufacturers' quota or export quota; and downstream issues such as export trading by pharmacies, speculative stockholding by pharmacies and stockpiling by patients in case of anticipated shortages e.g. UK exit from Europe (Brexit). Alternatively, some barriers were reported to be within the WDs' control to ameliorate. These included: the use of generics (which the WD procures); and WD decision to limit supplies to pharmacies through quotas (where supplies from manufacturers were limited, quotas were used to equitably share supply).

#### 5.3.1 Supply chain disruptions

Manufacturers' commercial decisions on where to send their product worldwide (influenced by UK regulations, medicines pricing and the value of sterling) impacted supply. These commercial decisions, together with globalisation of manufacturing sites, meant WDs could have limited supplies of medicines, leaving them unable to meet customer demand.

Many of the WDs referred to shortages impacting their ability to supply customers. Shortages and medicines out of stock at manufacturer sites required WDs to respond by sourcing alternatives and increasing stockholdings of other products. WDs expressed concern about supply assurance regarding palliative medicines.

*“…We're concerned…that manufacturers will…choose not to supply the drugs to the UK because they will be able to make more money supplying it elsewhere in the EU. So…the supply chain…is a really big worry and for palliative medicines that's especially important. This isn't something where you can order something in and wait 2 months…it's being ordered because it is needed there and then and once you've missed that chance to support the patient at that crucial moment in their lives that moment has gone…”* (WD06, LM).

#### 5.3.2 Strategic drivers

Strategic supply influences that acted as barriers included: generic medicines, quotas and storage capacity, all of which influenced WDs' decision-making. WDs identified the supply of generics as a commodity market, in contrast to the branded medicines channel. High demand for generics was perceived as created through NHS commissioning/incentive schemes as well as community pharmacy purchasing. In contrast, non-availability of a generic was viewed as the result of low demand worldwide or low profit margins, leading manufacturers to withdraw the product from the market.

Most participants referred to introducing quotas to ration medicines and assure equitable distribution. Supply quotas could be put in place by Governments in other countries, limiting parallel importation of medicines (such as branded medicines brought in from other European countries to sell at a higher price in the UK). Product quotas could be put in place by WDs to prevent over-ordering and trading by pharmacies.

Some participants asserted that WDs' storage capacity for secure and temperature-controlled medicines could impact on the supply chain. This was perceived to be a barrier in relation to the volume and space for storage of palliative care CDs:

*“…Because we don't want too much stock particularly when we are talking about controlled drugs of course there is a limitation on how much space you can actually have...”* (WD03, FL).

#### 5.3.3 Downstream issues

Participants reported instances of downstream issues which affected the supply chain. These included export trading by pharmacies; product switches; geographical differences in palliative medicines lists; speculative stockholding by pharmacies; changes in prescribing habits and stockpiling by patients due to lack of understanding about the supply chain.

#### 5.3.4 Upstream issues

The inability of manufacturers to adequately predict operational issues or forecast demand led to production issues and shortages of manufactured stock. WDs considered the notice period and delay in release of information from manufacturers/Department of Health and Social Care, reportedly due to commercial sensitivity, problematic. They considered there was not always enough time to make alternative arrangements e.g. order in alternative products to maintain supplies into pharmacies and endeavoured to work closely with manufacturer account managers to assure product availability.

Product recalls also undermined confidence in specific products and the supply chain. Manufacturing errors caused an increase in pharmacy workload via recovering specific batched products and re-dispensing new products (same or alternatives) which could lead to temporary medicines shortages, reducing access to medicines.

## 6 Summary of facilitators and barriers to supply

The findings highlight issues affecting access to medicines used during the last year of life, as relayed by two major stakeholders in the pharmaceutical supply chain, CPs and WDs. Table 2 provides a summary of facilitators and barriers to supply to community pharmacy as perceived by these groups.

**Table 2:** Facilitators and barriers to supply into community pharmacy.

Community pharmacist findings		Wholesaler/distributor findings	
Facilitators (+)	Barriers (−)	Facilitators (+)	Barriers (−)
• *Relationship building* – with patients, families and myriad of relevant healthcare professionals (embedding in communities and local healthcare services) • *Use of key wholesalers/distributors* – 1-2 WDs used as first-line options, protocol driven prioritisation of WDs (which to use when). • *Online information technology systems for stock management and ordering* • *Time to delivery* – same or next weekday delivery, multiple deliveries per day via multiple WDs. • *Sourcing stock from other pharmacies* – networks run via instant messaging apps.	• *Medicine shortages* – a universal challenge, related requirement to seek out information via professional organisations. Lead to quotas (rationalisation), price rises and last resort a prescription change. • *Need to use multiple WDs* – creates complexity (multiple supply routes) and onerous workload. Required because of Solus agreements and medicine shortages. • *Lack of communication and relationships with WDs and manufacturers* – lack of meaningful two-way communication underpinned by trust with WDs and manufacturers, consequent lack of relationships. CP contact with WDs via telesales service centre staff, with no clinical insight and reliance on information technology systems. • *Shortcomings of ordering systems* – despite the systems being a facilitator to supply, they could also act as a barrier as: systems not sufficiently live, CPs needed to phone WDs to try to find out information on a vast array of issues e.g. when a product would be back in stock, how long a product would take to be delivered if it had been switched to a different warehouse, brand availability, expiry dates. • *Disincentives to stock palliative medicines* – for some CPs lack of stock turnover of such medicines (associated costs and lack of long shelf life), added issues with controlled drugs (e.g. need to store in locked cupboard, inability to return controlled drugs to WDs). • *Lack of weekend ordering and Sunday deliveries* – requirement to wait for Monday's deliveries led to problematic supply over weekends. • *Issues with WD deliveries* – medicines missing from the delivery (usually when the medicines had become out of stock at the WDs, but CPs did not know this until the delivery arrived), occasional other issues e.g. delivery drivers doing too many hours (so return to base without making deliveries).	• *Relationship-building* – importance of methods, contractual/informal communications and creation of feedback channels. • *Upstream relationships with manufacturers/suppliers* – contractual relationships, information-sharing, supply certainty. • *Downstream relationships with pharmacies* – responsibility to assure supply, timely information, middleman position. • *Collaborative relationships* – collaborative good practice, working to common agenda, patient safety, roles of other parties. • *Investment in logistics infrastructure* – choice of partners, development of logistics equipment, impact on service responsiveness. • *Demand and stock management* – access to stock pools, supply continuity, impact of generics, sharing of demand patterns (WD and pharmacies). • *Buffer stock availability* – in UK and Western Europe, additional resilience in the supply chain, changing roles of full and short-line wholesalers during stock droughts.	• *Supply disruptions* – United Kingdom regulations, pricing and value of sterling; medicine shortages. • *Strategic drivers* – demand for generics, quotas and WD storage capacity. • *Downstream issues* – export trading by pharmacies, geographical differences in palliative care medicine lists, speculative stockholding by pharmacies, stockpiling by patients. • *Upstream issues* – inadequate forecasting, prediction of operational issues, manufacturing shortages, lack of timely information, product recalls, reputational damage, temporary stock shortages.

Abbreviations: WD – wholesaler/distributor; CP – community pharmacist.

## 7 Discussion

As globalisation of manufacture increases shortages are more likely,^35^ coupled with increasing demand for supplies of palliative care medicines in the community due to ageing populations and more people wanting and actually dying at home,^36^ challenges sustaining supplies will persist. Key issues raised in our findings, via two hard-to-reach sample groups were: the impact of medicines shortages; supply chain performance issues; and lack of information sharing via effective relationships; each is discussed in turn with recommendations suggested.

### 7.1 The impact of medicines shortages

Lack of access to medicines was found to be problematic within the pharmaceutical supply chain and caused strain on community pharmacies. Palliative medicines supply into community pharmacy was perceived by CPs to function satisfactorily; except when unable to get hold of supplies due to products being unavailable (similarly noted by Kuruvilla et al^29^ relative to palliative medicines). Medicines shortages therefore led to additional work for CPs to access products for patients via multiple routes and systems, and it was their professional obligation to do this work that masked inherent difficulties. Whilst they had a preferred WD (primary wholesaler, also demonstrated in the work of Sengun & Wasti^37^) who they worked closely with, they engaged with numerous WDs to gain supplies of medicines, endlessly having to shop “*around from one to another*” (CP14, I) to do so. The efforts of CPs in accessing medicines propped up a fragile supply chain at times when medicines were in short supply. They were doing their *“damnedest”* to get medicines for patients (CP15, I). Studies focusing on hospital pharmacists and technicians underline the additional workload incurred by product shortages, via having to investigate and source alternatives.^15^^,^^32^ Careful consideration of skill mix in the pharmacy will help release CPs from some of this additional workload but there needs to be recognition that not all such activity can be successfully delegated as CPs' clinical insight and knowledge may be required. The relative success of skill mix alterations will depend on NHS funding and pharmacy chain/contractor investment in staff and education.

One of the main problems reported by WDs (like CPs) was medicines shortages from manufacturers perceived to be outside the WDs' control, despite sharing information on product demand. Supply disruptions and shortages can lead to WD reputational damage.^32^ However, despite having sophisticated materials management systems to secure stock and fulfil customer orders, WDs reported common-place downstream issues such as trading by pharmacies, regional or local product switches and stockpiling of drugs (by pharmacies and patients), causing problems. These led to enforcement of stock restrictions via quotas, to ration medicines supplies and restrict these activities, limiting access to medicines. CPs navigated a challenging interface with, and often competing pressures between: WDs (via customer service centres); the Department of Health and Social Care (via the Drug Tariff) to ensure cost reimbursement of medicines; and multiple systems (e.g. regulatory, legal, contractual, organisational and IT based). In doing so they were mindful of their duty to patients despite competing ethical and commercial pressures (pressures highlighted by other researchers in relation to professional duty of care and autonomy vs retail pressures of open markets, monopolies and economic autonomy^38^^,^^39^). Professional diligence compounded the onerous levels of work undertaken, which has also been observed as exacerbated in the face of shortages in other contexts, such as for hospital pharmacists and GPs.^40^^,^^41^ Crucially, this level of work may detract from CPs embracing wider patient-facing roles in palliative care, for example as palliative medicines' information-giving specialists as advocated by Royal Pharmaceutical Society Wales,^24^ and could cause further role conflict.^39^

### 7.2 Supply chain performance issues

In business, alliances between trusted partners are critical, reducing vulnerabilities and risk thereby offering greater capacity and capability. For example, direct alignment with a WD means supply into community pharmacy should be assured across key product lines (e.g. Pfizer's partnership with Alliance Healthcare WD and AstraZeneca's with AAH WD). Partnerships need to be purposeful, with the right partners who are loyal and committed.^42^^,^^43^ Sinkovics et al^44^ reported collaborations to be an issue and these were raised in our study as an area of weakness and ineffectiveness, impacting on medicines availability. Stakeholders within the pharmaceutical supply chain work together to a common agenda and as such there is an innate dependency on each other, but levels of influence and power can shift depending on the stakeholder relationship and situation.^18^,^45^ Access to palliative medicines in this situation had left CPs regarding themselves as being “*at the end of the chain*,” unable to get hold of products at the beginning of the chain. This put CPs in a vulnerable situation and has been experienced in other contexts.^45^ WDs partnered with manufacturers, acting in an intermediary capacity^46^ to source stocks and ensured availability of these with numerous regional storage locations/distribution centres. In the current climate, when considering medical supply chains, regional holding of stock is considered essential to enable supply chain responsiveness alongside public-private partnerships e.g. the NHS and pharmaceutical manufacturers and optimal stock holding levels.^6^

Overall, CP views of the performance of the pharmaceutical supply chain into community pharmacy and availability of palliative medicines were negative. Practices that were put in place to facilitate stronger supply routes: from the manufacturer (Solus agreements); reimbursement systems (Drug Tariff) and equitable supply (quota systems); paradoxically appeared to increase the risk of delays and/or shortages. Sengun & Wasti^37^ when discussing pharmacy/wholesaler alliances, stated that these partnerships benefit from competency in role, goodwill, the ability to manage conflict resolution, trust and a level of formal control; some of which appeared lacking in the relationships explored in our study. CPs were critical of supply chain processes, perceiving them as a series of challenging hurdles that needed to be circumnavigated. All of which had the potential to undermine trust among supply chain stakeholders, confidence in the system itself and disruptions in medicines deliveries, leaving patients vulnerable; correspondingly demonstrated in the work of Bhaskar et al^6^ and Revilla et al.^47^

### 7.3 Lack of information sharing via effective relationships

Information sharing is critical in the pharmaceutical supply chain to effect responsiveness and share risks and benefits.^48^ CPs' ability to respond to medicine shortages were hampered by having no consistent source that informed them of shortages, they needed to collate relevant information from sources including pharmaceutical wholesalers, manufacturers, pharmacy press, other pharmacies, colleagues, and social media. Overall, WDs were aware of challenges in the supply chain, particularly meeting unexpected demand, and claimed to act as a point of liaison in the triadic relationship between manufacturer, WD and community pharmacy, which has been seen to be an expectation in this role elsewhere.^46^ They felt they managed relationships with manufacturers on behalf of CPs, as well as on their own behalf, as also found by Sinkovics et al.^44^ WDs viewed relationships with CPs as effective, despite CPs doubting the commercial motivations and actions of WDs (as also observed in the work of Wong et al^46^). In contrast, CPs argued where contact did occur with WDs (via telesales staff) this was not the result of productive or satisfactory relationships. Such contact was perceived as not that helpful, as the information transfer from WDs was only as good as the information on the IT system.

Improved information sharing regarding supply issues and shortages would benefit CPs and reduce unnecessary work. During COVID-19 information has been informally shared via multiple routes such as professional networks, email, and messaging services. Whilst this has been helpful greater centralisation of information via a single route would reduce work further. Key to further releasing CPs' time (alongside the skill mix considerations cited earlier) are: greater use of hub and spoke pharmacy, separation of supply and advice roles, electronic prescriptions and ordering processes all of which can be used to streamline the environments that pharmacies work in, so work is managed across all times of day.

### 7.4 A conceptual model of supply into community pharmacy

**Fig. 3:**
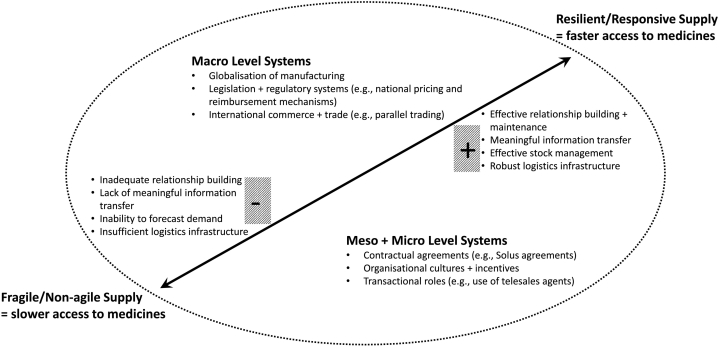
Conceptual model of supply into community pharmacy.

Bhaskar et al,^6^ when discussing medical supply chains during the COVID-19 pandemic, asserted further coordination, integration, and management of global supply chains are needed. This premise can equally be applied to global pharmaceutical supply chains, to build resilience via a well-functioning supply chain and reduce failures such as medicines shortages. In this study, the interplay of stakeholders at macro, *meso* and micro levels and influencing factors (positive and negative) were found to affect palliative medicines supply into community pharmacy. This inter-relationship is captured in a conceptual model representing and characterising the impact of supply of palliative medicines into community pharmacy as shown in Fig. 3. The model developed following data triangulation across the sample groups, analysing the factors facilitating speed of supply and the contextual systems that influence these factors.

The ‘whole system’ of supply was influenced by macro (legal, regulatory and economic external conditions nationally and internationally), *meso* (local organisational factors and influences such as organisational culture and incentives) and micro (individual interaction, both helped and hindered by IT) level systems. At the macro level increasing globalisation of pharmaceutical manufacturing and supply, amplified the likelihood of medicine shortages nationally, with supply additionally bounded by national pricing and reimbursement mechanisms.

Table 3 depicts a logic model which contextualises the impact (and mechanisms of impact) of activity undertaken at the macro, *meso*, and micro levels on palliative medicines availability. Each level interrelated via a cascade of effects, from the macro through the *meso* and micro levels. For example, national legislation and regulatory systems on pricing and reimbursement (macro level) framed and influenced ‘contractual’ agreements operating at the meso level (e.g. discount agreements WDs offered to CPs based on stock volumes purchased; prioritisation of WDs by community pharmacies based on medicine prices offered) and agreements influenced transactions at the individual (micro) level (e.g. the WD customer service centres CPs and dispensers need to interact with). As can be observed the interaction between mediating factors within the macro and meso levels was complex, both positively and negatively impacting the responsiveness of medicines supply. In contrast, at the micro level the interplay of transactional roles appeared to have a solely negative impact on supply, limiting responsiveness.

**Table 3:** A logic model depicting the impact of macro, meso and micro level system factors on medicines supply into community pharmacy.

*Macro* level systems	Mechanism	Positive influencing factors	Negative influencing factors	Proposed impact on supply
Globalisation of manufacturing	Increased outsourcing of manufacturing and resulting increases in global supply routes	• Skilled management of remote operations required • Robust logistics and pre-wholesale infrastructure required	~	More responsive medicines supply, where implementation of the influencing factors is skilled and robust
	Finite active ingredients/raw materials globally may limit production	~	• Global medicines shortages may be induced by limited active ingredients/raw materials • Insufficient capacity for manufacture via limited raw materials leads to shortages, and/or affects production schedule and lead times for production	Less responsive medicines supply
Legislation and regulatory systems	National pricing and reimbursement mechanisms via the Drug Tariff in England	~	• National retrospective reimbursement system may preclude manufacturers from navigating market entry requirements for medicines into the United Kingdom	Less responsive medicines supply
	Price concessions via the Department of Health and Social Care in England – increase reimbursement prices for certain medicines (for the month in which they are granted)	~	• Price concession levels not known at time of stock procurement by CPs, products returned to WD if price deemed too high or CPs accommodate a potential loss between purchase price and price concession	Less responsive medicines supply
	Influence of the Department of Health and Social Care in the case of severe shortages	• General willingness of WDs to work collaboratively in the case of severe shortages	• Any marketplace shortage drives up price of medicine • Lack of national level guidance on managing shortages except in the case of severe shortages • Potential prioritisation of hospital supply over community supply in the case of severe shortages	Impact depends on the balance of positive and negative influencing factors, but more responsive medicines supply is likely overall in the case of severe shortages
	Legislation surrounding supply of controlled drugs – requirements for locked storage, inability to return controlled drugs to WDs, requirements around destruction of out of date controlled drugs	~	• Controlled drugs related legislation acts as disincentive to stocking these medicines by CPs (requirement of locked cupboard storage and inability to return controlled drugs to WD), and limits storage capacity at WDs	Less responsive medicines supply
	Medicines regulatory agency ensures compliance with regulations	• Auditing of manufacturers by regulators; and WDs must maintain regulatory compliance	~	More responsive medicines supply
International commerce and trade	Global competition for medicines supply at competitive prices	~	• Difficulty forecasting demand for WDs and manufacturers • Insufficient product to meet overall global demand • Global low demand for generics leads to products being withdrawn from the marketplace	Less responsive medicines supply
	Parallel trading across countries	• WD ability to source products outside of the United Kingdom	~	More responsive medicines supply
	Withdrawal from the European Union (Brexit)	• Establishment of national stockpiles of key medicines for European Union withdrawal	~	More responsive medicines supply nationally, but may induce less responsive supply in other countries
	Quotas may be implemented by Governments in other countries	~	• International trade limited by quotas imposed abroad	Less responsive medicines supply

*Meso* level systems				
Contractual agreements	Contracting agreements between WDs and manufacturers	• Provide assurance of inbound stock to WDs	~	More responsive medicines supply
	Contracting agreements between WDs and haulage/logistics firms	• Helps to ensure delivery to CPs, with twice daily deliveries by full-line wholesalers	~	More responsive medicines supply
	Solus agreements - sole WD for some manufacturers	• Increased security and less fragmented supply chain	• Risk of supply failure as products cannot be accessed via other WDs • CPs required to use multiple WDs to accommodate Solus agreements, add complexity to supply chain routes	Impact depends on the balance of positive and negative influencing factors, but less responsive medicines supply is likely overall
	Prioritisation of WDs by community pharmacies – one WD used as first line, another as second line and so on	• Cascade protocols for CPs of which WDs to use enable supply decisions based on cost, availability and speed of access	• Cascade protocols for CPs add complexity to supply chain routes	Impact depends on the balance of positive and negative influencing factors, but more responsive medicines supply is likely overall
	WD discount agreements made with CPs based on volume of stock purchased	~	• Price discounts encourage larger pharmacies to bulk purchase and hold warehouse stocks of medicines	Less responsive medicines supply
Organisational cultures and incentives	Contrasting cultures between WDs and CPs	~	• Lack of meaningful two-way communication • Lack of relationship building • Mistrust on part of CPs of WDs motivations and actions	Less responsive medicines supply due to ineffective information sharing with upstream supply chain stakeholders (community pharmacies to manufacturers via WDs). Or less responsive medicines supply as manufacturers are not provided with direct feedback from CPs.
	Commercial priorities of WDs	~	• Lack of meaningful two-way communication • Lack of relationship building • Mistrust on part of CPs of WDs motivations and actions	Less responsive medicines supply
	Patient facing focus of CPs (accountability to the patient) but underlying commercial incentives for CPs	~	• Lack of meaningful two-way communication • Lack of relationship building • Mistrust on part of CPs of WDs motivations and actions	Less responsive medicines supply
Information technology stock management and ordering systems	Information technology systems facilitate sophisticated stock management by WDs (accounting for demand patterns, stock holding levels and locations of stock)	• Stock volumes at WDs managed across distribution centres/warehouses • Liaison with manufacturers based on recent demand	~	More responsive medicines supply, except where an increase in demand exceeds recent demand
	Information technology systems generally facilitate CP ordering and time to delivery	• WD deliveries Mon-Sat	• CP orders Mon-Fri only • Cut-off times for CP ordering must be met for same or next day delivery • Ordering systems may be insufficiently live or be limited in functionality	Impact depends on the balance of positive and negative influencing factors, but more responsive medicines supply is likely overall

*Micro* level systems				
Transactional roles	Use of telesales agents (lack of clinical insight/understanding of palliative medicines)	~	• Lack of relationship development between CPs and WDs • Lack of feedback received by WDs from patients and carers/CPs • Lack of meaningful two-way information transfer between CPs and WDs	Less responsive medicines supply
	Delegation of customer service interaction with WDs to dispensers in community pharmacy	~	• Lack of relationship development between CPs and WDs • Lack of feedback received by WDs from patients and carers/CPs • Lack of meaningful two-way information transfer between CPs and WDs	Less responsive medicines supply
	Information transfer from WD customer centres to CPs limited to that contained within the information technology system	~	• Lack of meaningful two-way information transfer between CPs and WDs	Less responsive medicines supply

Abbreviations: WD – wholesaler/distributor; CP – community pharmacist.

Within the context of macro, meso and micro level systems, effective relationship-building, meaningful information transfer, effective stock management and robust logistics infrastructure lead to a resilient and more responsive supply chain, potentially enabling faster medicines access for patients at EoL.

Given the influence of the globalisation of medicines manufacturing (a macro level system) on supply into community pharmacy, the COVID-19 pandemic has underlined that shortages of palliative medicines, such as alfentanil, and medicines more widely^49^, ^50^, ^51^, 52. will continue. Therefore, development of resilient supply chains which secure access to palliative medicines is crucial. To this end macro level manufacturing and logistics practices are required to prevent medicines shortages e.g., investment in national manufacturing capability. Micro level systems such as individual transactional roles which influence the supply chain are perhaps easier and less costly, to change. Therefore, a focus on developing and implementing methods to promote relationship development (between WDs and CPs) and meaningful two-way information transfer would be pragmatic. Such a focus would facilitate patient and carer feedback on medicines supply up the supply chain.

## 8 Strengths and limitations

This is the first study investigating the views of community pharmacists and wholesalers/distributors on supply into community pharmacy of palliative medicines. Supply of these medicines into community pharmacy will continue to be critical going forward due to the likelihood of a sustained increase in those dying at home over the next decade. Despite both sample groups being hard-to-reach and recruit (CPs were difficult to contact, and workloads meant that to participate the majority did so in their own time), sufficient participants were recruited to gain rich, detailed perspectives.

Study limitations were the non- involvement of manufacturers of palliative medicines in the study, the small sample size of WDs and the timing of data collection. Data were collected in 2019, so views shared were impacted by Brexit, but this facilitated shortage issues and the supply chain to be brought into sharp focus, just prior to the impact of COVID-19. Accessing WD participants was extremely problematic; some were only accessible by account holders; invitations to participate were made several times (via multiple routes including directly and via the trade association) with no response. This may have been due to the work pressures associated with preventing medicines shortages surrounding Brexit; political sensitivities about medicines shortages; and commercial sensitivities. Nevertheless, we managed to secure important insights from a group of WDs serving different elements of the supply chain. WD perspectives are seldom included in supply chain research and therefore this study presents an important, unique perspective. The respondent profile in the study was weighted in favour of CPs, as the potential sample pool was significantly greater than for WDs, enabling data saturation to be achieved for this sample.

Future research in this field should extend to interviewing manufacturers and examination of alternative models of community supply of palliative medicines such as locality-based hubs (e.g. within Acute Trusts or Out-of-Hours centres facilitating 24/7 access to pharmacists and required medicines) or through rapid response teams.

## 9 Conclusions

The aim of this study was to identify community pharmacists' and pharmaceutical wholesalers'/distributors' views on supply chain processes and challenges in providing access to medicines during the last year of life in the UK context. The study demonstrated for the first time the issues community pharmacies encounter in relation to access to palliative medicines. A conceptual model for supply into community pharmacy resulted, demonstrating the complex interplay between influencing factors and the effect on responsiveness of supply and ultimately speed of medicines access for patients. This has the potential to be used by practitioners, policy makers, wholesalers/distributors, and researchers to inform and evaluate changes to improve the pharmaceutical supply chain for all medicines but particularly palliative medicines. The study also highlighted the vital role community pharmacists and pharmaceutical wholesalers/distributors play in ensuring access, and community pharmacists' willingness to undertake arduous and time consuming ‘work-arounds' to navigate the supply chain to gain products for patients. Future work is required to integrate and manage this supply chain, facilitating effective relationship-building and essential information-sharing between stakeholders. With this in place patients will be less likely to endure the impact of medicines shortages and disrupted access at EoL, and community pharmacists will be freed up to undertake the extended roles in this area advocated by professional bodies.

## Funding acknowledgement

This study was funded by the National Institute for Health Research (NIHR) [Health Services & Delivery Research programme], UK (project number 16/52/23). The views expressed are those of the authors and not necessarily those of the NIHR or the Department of Health and Social Care.

Professor Richardson is a 10.13039/501100000272National Institute for Health Research (NIHR) Senior Investigator. The views expressed in this article are those of the author and not necessarily those of the NHS, the NIHR, or the 10.13039/501100003921Department of Health.

## Author contributions

NC, SL, AR, MB and JB contributed to the concept and design of the work; NC and EM acquired all data; NC, EM, LB and SL contributed to data analysis; NC and LB drafted the article; all authors revised it critically for intellectual content; all authors have participated sufficiently in the work for appropriate portions of the content. All authors approved the submission of the manuscript for publication.

## Declaration of Competing Interest

The authors declare that they have no known competing financial interests or personal relationships that could have appeared to influence the work reported in this paper.

## References

[1] Healthcare Distribution Alliance (2020). https://www.hda.org/about/role-of-distributors.

[2] Breen L. (2008). A preliminary examination of risk in the Pharmaceutical Supply Chain (PSC) in the National Health Service (NHS). J Serv Sci Manag.

[3] Breen L., Hou J., Sowter J. (2021). Advancing the understanding of pharmaceutical supply chain resilience using Complex Adaptive System (CAS) theory. Supply Chain Manag.: an Int. J..

[4] Narayana S.A., Pati R.K., Vrat P. (2014). Managerial research on the pharmaceutical supply chain–A critical review and some insights for future directions. J Purch Supply Manag.

[5] Rossetti C.L., Handfield R., Dooley K.J. (2011). Forces, trends, and decisions in pharmaceutical supply chain management. International Journal of Physical Distribution & Logistics Management.

[6] Bhaskar S., Tan J., Bogers M.L. (2020). At the epicenter of COVID-19–the tragic failure of the global supply chain for medical supplies. Front Public Health.

[7] Priyadarshini R., Jafrin L., Aravinthan A., Sivagnanam G. (2021). Rationing PPEs during a pandemic: the COVID-19 scenario. Research in Social & Administrative Pharmacy.

[8] Beck M., Buckley J., O’Reilly S. (2019). Managing pharmaceutical shortages: an overview and classification of policy responses in Europe and the USA. Int Rev Adm Sci.

[9] Claus B., Pauwels K., Baert M. (2015). Drug shortages in the hospital: management, causes and budget impact. J Pharm Belg.

[10] De Weerdt E., Simoens S., Hombroeckx L., Casteels M., Huys I. (2015). Causes of drug shortages in the legal pharmaceutical framework. Regul Toxicol Pharmacol.

[11] Fox E.R., Sweet B.V., Jensen V. (2014). Drug shortages: a complex health care crisis. Mayo Clin Proc.

[12] Kaakeh R., Sweet B.V., Reilly C. (2011). Impact of drug shortages on US health systems. Am J Health Syst Pharm.

[13] Miljković N., Gibbons N., Batista A., Fitzpatrick R.W., Underhill J., Horák P. (2019). Results of EAHP’s 2018 survey on medicines shortages. Eur J Hosp Pharm.

[14] Pauwels K., Simoens S., Casteels M., Huys I. (2015). Insights into European drug shortages: a survey of hospital pharmacists. PLoS One.

[15] Rosoff P.M., Patel K.R., Scates A., Rhea G., Bush P.W., Govert J.A. (2012). Coping with critical drug shortages: an ethical approach for allocating scarce resources in hospitals. Arch Intern Med.

[16] Said A., Goebel R., Ganso M., Zagermann-Muncke P., Schulz M. (2018). Drug shortages may compromise patient safety: results of a survey of the reference pharmacies of the drug Commission of German Pharmacists. Health Policy.

[17] World Health O (2016). Medicines shortages: global approaches to addressing shortages of essential medicines in health systems. WHO Drug Information.

[18] Connelly D., Cotterell M. (2019). Measuring the market. Pharm J.

[19] Pharmaceutical Services Negotiating Committee (2020). http://psnc.org.uk/psncs-work/about-community-pharmacy/.

[20] NHS Digital (2020). https://digital.nhs.uk/data-and-information/publications/statistical/general-pharmaceutical-services.

[21] Walter E., Dragosits A., Said M. (2012). Access to pharmaceutical products in six European countries–analysis of different pharmaceutical distribution systems. Farmeconomia Health economics and therapeutic pathways.

[22] European Association of Pharmaceutical Full-line Wholesalers (2015). http://girp.eu/sites/default/files/documents/the_role_of_pharmaceutical_full-line_wholesaler_081015.pdf.

[23] Walter E., Lazic-Peric A. (2017). http://girp.eu/files/GIRP-IPF%20Study%202016.pdf.

[24] Royal Pharmaceutical Society Wales Palliative & End of Life Care: Pharmacy's contribution to improved patient care. RPS Wales. https://www.rpharms.com/Portals/0/RPS%20document%20library/Open%20access/Policy/RPS%20Wales%20Palliative%20and%20End%20of%20Life%20Care%20Policy%20WEB.pdf?ver=2019-06-17-120534-630.

[25] Sleeman K., Murtagh F., Kumar R. (2021). Better end of life 2021: dying, death and bereavement during Covid-19 research. Research Report, London..

[26] Ogi M., Campling N., Birtwistle J. (2021). Community access to palliative care medicines—patient and professional experience: systematic review and narrative synthesis. BMJ Support Palliat Care.

[27] Lucey M., McQuillan R., MacCallion A., Corrigan M., Flynn J., Connaire K. (2008). Access to medications in the community by patients in a palliative setting. A systems analysis. Palliat Med..

[28] Miller E.J., Morgan J.D., Blenkinsopp A. (2019). How timely is access to palliative care medicines in the community? A mixed methods study in a UK city. BMJ Open.

[29] Kuruvilla L., Weeks G., Eastman P., George J. (2018). Medication management for community palliative care patients and the role of a specialist palliative care pharmacist: a qualitative exploration of consumer and health care professional perspectives. Palliat Med.

[30] Latter S., Campling N., Birtwistle J. (2020). Supporting patient access to medicines in community palliative care: on-line survey of health professionals’ practice, perceived effectiveness and influencing factors. BMC Palliative Care.

[31] O’Brien B.C., Harris I.B., Beckman T.J., Reed D.A., Cook D.A. (2014). Standards for reporting qualitative research: a synthesis of recommendations. Acad Med.

[32] Harvey W.S. (2011). Strategies for conducting elite interviews. Qual Res.

[33] Ritchie J., Spencer L., Bryman A., Burgess R.G. (1994). Analysing Qualitative Data.

[34] NHS Business Services Authority (2020). http://www.nhsbsa.nhs.uk/prescription-data/dispensing-data/dispensing-contractors-data.

[35] Bogaert P., Bochenek T., Prokop A., Pilc A. (2015). A qualitative approach to a better understanding of the problems underlying drug shortages, as viewed from Belgian, French and the European Union’s perspectives. PLoS One.

[36] Higginson I.J., Brooks D., Barclay S. (2021). Dying at home during the pandemic. Br Med J.

[37] Şengün A.E., Nazli Wasti S. (2007). Trust, control, and risk: a test of das and Teng’s conceptual framework for pharmaceutical buyer-supplier relationships. Group & Organization Management.

[38] Quay S. Balancing the ethical and commercial pressures of community pharmacy. The Pharmaceutical Journal Web site https://pharmaceutical-journalcom/article/opinion/balancing-the-ethical-and-commercial-pressures-of-community-pharmacy [Published 2018. Accessed 10.12.21].

[39] Davies J.E. (2013).

[40] De Weerdt E., De Rijdt T., Simoens S., Casteels M., Huys I. (2017). Time spent by Belgian hospital pharmacists on supply disruptions and drug shortages: an exploratory study. PLoS One.

[41] Bostock N. (2020). Medicines shortages drive up GP workload as prescribing costs surge £158m. GP Online.

[42] Brinkhoff A., Özer Ö., Sargut G. (2015). All you need is trust? An examination of inter-organizational supply chain projects. Prod Oper Manag.

[43] Wu L., Chuang C.-H., Hsu C.-H. (2014). Information sharing and collaborative behaviors in enabling supply chain performance: a social exchange perspective. Int J Prod Econ.

[44] Sinkovics R.R., Jean R.-J.B., Roath A.S., Cavusgil S.T. (2011). Does IT integration really enhance supplier responsiveness in global supply chains?. Manag Int Rev.

[45] Kelliher F. (2007). Small firm cooperative constructs: addressing industry power relationships. J Small Bus Enterp Dev.

[46] Wong H.H., Io U.M., Hu H. (2012). Pharmaceutical wholesalers Service in Macau: an investigation from the perspective of stakeholders. Business and Management Research.

[47] Revilla E., Saenz M.J. (2017). The impact of risk management on the frequency of supply chain disruptions. Int J Oper Prod Manag.

[48] Cao M., Zhang Q. (2011). Supply chain collaboration: impact on collaborative advantage and firm performance. J Oper Manag.

[49] Alexander M., Jupp J., Chazan G., O’Connor S., Chan A. (2020). Global oncology pharmacy response to COVID-19 pandemic: medication access and safety. J Oncol Pharm Pract.

[50] Badreldin H.A., Atallah B. (2020).

[51] Newton P.N., Bond K.C., Adeyeye M. (2020). COVID-19 and risks to the supply and quality of tests, drugs, and vaccines. Lancet Glob Health.

[52] Romano S, Galante H, Figueira D, Mendes Z, Rodrigues AT. Time-trend analysis of medicine sales and shortages during COVID-19 outbreak: Data from community pharmacies. Research in Social and Administrative Pharmacy Web site. 10.1016/j.sapharm.2020.05.024. Published 2020. Accessed 7.11.20. PMC7245321 32482587

